# Ethical insights into AI-driven caries detection: a scoping review

**DOI:** 10.1038/s41405-025-00366-0

**Published:** 2025-09-09

**Authors:** Tahoora Yousuf, Madiha Khan, Robia Ghafoor

**Affiliations:** Operative Dentistry & Endodontics, Department of Surgery, Aga Khan University Hospital, Karachi, Pakistan

**Keywords:** Dental radiology, Diseases

## Abstract

**Background:**

Artificial Intelligence (AI) has become increasingly integrated into dental diagnostics, particularly for detecting carious lesions. While AI offers benefits such as improved accuracy and efficiency, its use raises important ethical concerns, including transparency, patient privacy, autonomy, diversity and accountability. This scoping review aims to identify these ethical concerns using a structured ethical framework.

**Methodology:**

Three databases were searched for papers regarding caries detection using AI. An established ethical framework was used to screen each paper for potential areas of concern.

**Results:**

A total of 351 abstracts were screened, of which 7 articles were included in this review. Each article was screened for established ethical principles including transparency, diversity, wellness, autonomy, privacy, accountability, equity, prudence, sustainable development, solidarity and governance. Diversity was the main ethical concern. Concerns related to accountability, equity and transparency were identified in 2 of the articles whereas ethical issue of privacy was identified in 4 of the articles. Only one study mentioned that no ethical approval was taken prior to commencement of study.

**Conclusion:**

AI in caries detection faces ethical issues like data bias, privacy risks, and equity concerns, potentially leading to flawed AI models. These issues can be addressed by creating a more specialized ethical framework that is specific to AI in dentistry.

**Clinical relevance:**

Understanding ethical challenges in AI-driven caries detection is critical to ensure accurate diagnostics, maintain patient trust, protect privacy, and support informed decision-making. Clinicians must be equipped to navigate these challenges as AI tools become more prevalent in dental practice.

## Introduction

Recent advancements in technology in the field of artificial intelligence (AI) have raised many hopes and concerns in health care, particularly in dentistry [1]. AI is a computer-based process that aims in healthcare transformation, including replacing physician responsibilities, improving patient-centered care to offer faster and more accurate diagnoses, and optimizing workflow and administrative activities [2]. It has various applications ranging from robotics to machine learning (ML) to other deep learning systems [2]. The majority of these applications employ ML in which machines learn human tasks without being explicitly taught [3].

In dentistry, dental image analysis utilizing AI has been demonstrated to be beneficial in detecting or classifying oral mucosal lesions, dental implant kinds, dental caries, and cephalometric landmarks, for example, with diagnostic accuracies comparable or superior to that of experts [4]. In the last 5 years the number of studies incorporating AI have rapidly expanded and majority of them concluded promising results with the utilization of AI in the detection of carious lesions using bitewing radiographs and intraoral photographs [1, 5, 6]. Interestingly, the detection of carious lesions using radiographs and photographs is defined differently. Radiographs detect carious lesions by identifying gradients of radiolucent areas, indicative of demineralization and decay within the tooth structure, including interproximal and subsurface regions [7]. This enables the detection of hidden and early-stage caries, offering a detailed view of the lesion’s depth and extent [7]. Conversely, photographs detect carious lesions based on visible surface changes, such as discoloration, pits, and cavities [8]. This is done through the International Caries Detection and Assessment System scoring criteria, which assess the extent and severity of the lesions by evaluating visual signs on the tooth surface [9]. A study by Kuhnisch et al. using intraoral photographs concluded that an AI model was able to correctly detect caries in 92.5% of cases [5]. Another study by Moran et al. conducted on bitewing radiographs also showed promising results and concluded that AI is an effective tool in detection of carious lesion severity [10]. Although the  literature appears to be hopeful  regarding  AI solutions for dentistry, all of these aspirations have also highlighted significant ethical concerns such as safety, equity and accountability [11]. While some attempts to engage in these ethical discussions have developed in recent years, the medical community remains unaware of some of other ethical complexities such as prudence, transparency and diversity that emerging AI technologies can pose [12, 13].

A recent systematic review by Tang et al. in the field of medical AI ethics was conducted to assess and bridge the gap between theoretical frameworks of AI ethics and the real expressed anxieties and concerns of current and prospective medical AI stakeholders, aiming to align these insights with an established ethical checklist [14, 15]. However, the literature on the ethical implications of AI used in dentistry for the detection of carious lesions, in accordance with an ethical framework remains scarce. The scarcity in literature is potentially concerning considering the possible influence of these technologies, because each practitioner is expected to advise their patients about the issues and hazards of a medical decision in order for them to make an informed decision [16]. Therefore, the aim of this scoping review is to identify ethical considerations associated with utilization of AI technology in the detection of carious lesions, aligning these concerns with an established ethical framework.

## Methodology

### Study design

Online databases such as PubMed, the Cochrane library, and Google Scholar were searched for all publications related to the ethical implications of the use of AI in dentistry, using the following keywords in a systematic manner:

(ethics OR ethical OR bioethics OR bioethical OR autonomy OR justice OR informed consent OR data management OR nonmaleficence) AND (artificial intelligence OR AI OR ML OR deep learning OR algorithms OR CNN[Convoluted Neural Network]) AND (dent OR dental OR caries OR caries detection OR endodontics OR decayed OR cavity OR dental radiograph) AND (clinical trials OR clinical OR trials OR randomized clinical trials)

### Inclusion criteria

Articles with the following features were included:

- Articles evaluating caries detection using photographs or radiographs, using artificial intelligence from 2015–2024
- Type of publications: Clinical trials, randomized clinical trials (RCT), experimental studies

### Exclusion criteria

- Studies addressing ethical challenges of AI in medical and health care without reference to ethical concerns in the field of dentistry
- Questionnaire based studies (evaluating perception and knowledge of AI ethical concerns)
- Review articles
- Publications not in English

### Search strategy

**Fig. 1:**
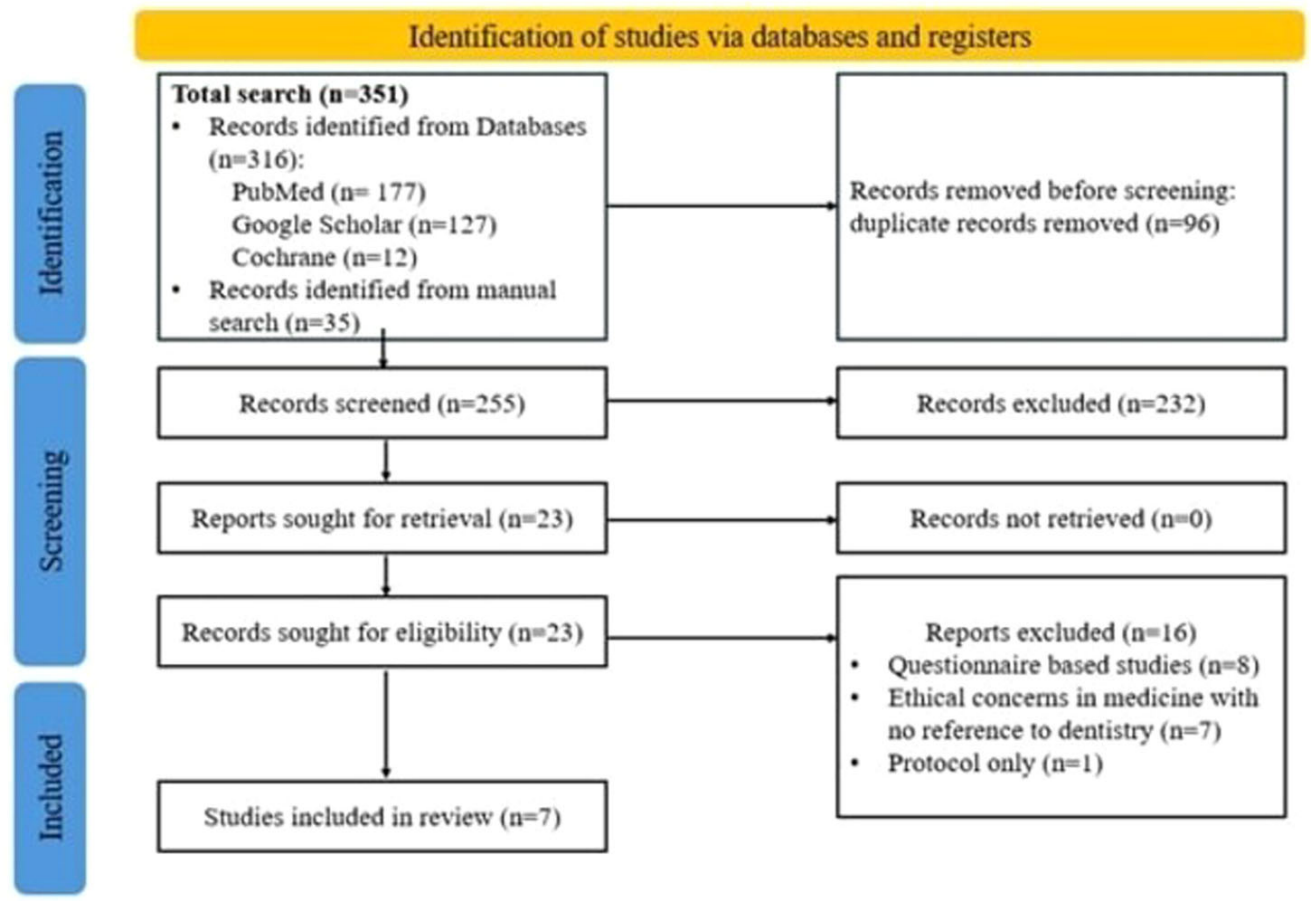
PRISMA Flowchart.

Using the aforementioned search strategy, articles were screened by two authors independently and a third author was consulted in case of disagreement. A PRISMA (Preferred Reporting Items for Systematic Reviews and Meta-Analyses) flowchart was formulated for the literature search and selection (Fig. 1). The screening and selection of publications was divided into two stages: an initial screening of titles and abstracts, followed by a final evaluation of full text publications. In the initial screening phase, article titles were screened for relevance, which was followed by reviewing of the abstracts. After the initial screening phase, both authors carried out a full text evaluation, considering the aforementioned inclusion and exclusion criteria. A third author was consulted in case of any disagreements.

### Framework for ethical considerations

Once the relevant articles were identified, each article was evaluated for potential ethical concerns by two authors. This was carried out by using a framework for ethical considerations, as developed by Rokhshad et al. [15]. The checklist was formulated by reviewing existing guidance documents on the ethical aspects of AI in healthcare from the World Health Organization and a recent scoping review on ethics reporting in dentistry [1]. The steering committee identified ten key items: diversity, transparency, wellness, privacy protection, solidarity, equity, prudence, sustainable development, accountability and responsibility, respect for autonomy, and decision-making [15]. These items were then discussed, revised, or expanded upon through one-on-one interviews with 29 members of the Topic Group Dental Diagnostics and Digital Dentistry, ITU/WHO (International Telecommunication Union/ World Health Organization) Focus Group AI on Health, and 3 AI ethics experts [15]. The framework emphasizes eleven key components, namely: Transparency, Diversity, Wellness, Respect of autonomous decision-making, Protection of privacy, Accountability and responsibility, Equity, Prudence, Sustainable development, Solidarity and Governance.

To ensure its relevance to AI-driven caries detection, we applied this framework by aligning each selected study with these eleven key ethical components. The key components of each aspect have been described in Table 1. For each article, ethical concerns were identified and mapped to these predefined categories.

**Table 1:** Key components of the ethical framework for AI in dentistry [15].

Transparency	Comprehensive documentation of AI models is essential, including clear and accessible descriptions of the inclusion and exclusion criteria for training and testing data. Patients should be informed about the use of AI in clinical practice.
Diversity	Datasets should represent the intended population and avoid bias related to ethnicity, age, gender, or health status.
Wellness	AI systems should promote the well-being of patients, clinicians, and healthcare organizations.
Respect of autonomous decision-making	AI should empower patients to maintain control over their care and environment, without diminishing their role in decision-making.
Protection of privacy	AI development must follow local data protection laws and safeguard against misuse of collected data.
Accountability and responsibility	Responsibility for AI-assisted decisions should be shared between clinicians and patients.
Equity	AI should promote fairness and equity and avoid exacerbating existing disparities.
Prudence	AI use in dentistry demands expertise and thoughtful application; clinicians should have the necessary digital literacy to interpret and act on AI outputs.
Sustainable development	AI should align with WHO Sustainable Development Goals, with resources evaluated for sustainability benefits in dental care.
Solidarity	AI should encourage cooperation and solidarity among all stakeholders in healthcare.
Governance	AI development and application in dentistry should comply with relevant regulations and oversight mechanisms.

During the review process, the two authors independently categorized each study based on the framework, classifying ethical concerns as explicitly addressed, partially addressed, or not addressed within the study. In case of any disagreements, a third author was consulted.

To enhance the depth of analysis, we also examined how these principles were operationalized—for example, whether transparency involved model explainability tools or if privacy was ensured via anonymization techniques. We further noted gaps, such as the absence of informed consent or unclear accountability, and qualitatively assessed their severity in terms of risks like data misuse, diagnostic bias, or erosion of patient trust. This structured approach enabled a more nuanced understanding of ethical integration in AI-based caries detection research.

### Ethical considerations

Ethical approval and consent was not required for the systematic assessment of articles as no human participants or personal data were involved.

## Results

### Identification of articles on ethical implications with the use of AI in dentistry

Using the described search strategy, a total of 351 abstracts were considered, which were reduced to 255 abstracts after removal of duplicate articles. These 255 articles were screened for titles and abstracts, with 23 being chosen for full text screening. Furthermore, 8 publications were excluded based on their study design (questionnaire-based study), and another 8 studies were excluded based on protocol and literature mentioning ethical concerns in other health care related fields with no reference in dentistry. As a result, a total of 7 publications were selected for this review. This is illustrated in (Fig. 1).

### Characteristics of included studies

All the studies included in this review showed that diversity was the main ethical concern as seen in studies by Mertens et al., Kuhnisch et al., Lee et al., Berdouses et al., Barayan et al., Moran et al., and Asker et al. acknowledging challenges related to representation in AI model development [5, 6, 10, 17–20]. Concerns related to accountability were identified in studies by Kuhnisch et al. and Moran et al, while ethical principles such as transparency, patient autonomy, and governance were largely absent from the discussions in the reviewed literature [5, 10]. A summary of included publications including study design, sample size, methodology, ethical concerns and the conclusion is presented in (Table 2).

**Table 2:** Publications regarding ethical concerns of AI used in caries detection.

S.No	Author (year)	Study design; Sample size (*n*)	Mode of carious detection	Methodology	Ethical concerns
1	Berdouses et al. [19]	Experimental study; (*n* = 425 regions of interest from 103 photographs from 91 extracted permanent teeth + 12 in vivo human teeth)	Intra oral Photographs	Algorithms (RF, RT, J48, SVM, and NB) were used for the assessment of carious lesions on the occlusal surface of permanent teeth using photographs	• No ethical approval • Diversity (single-centered study) • Equity (Difficulty bias) • Protection of privacy
2	Mertens et al. [20]	RCT; (*n* = 140 Bitewing radiographs of permanent teeth)	Bitewing radiographs (BW)	Proximal caries detection by dentists with and without CNN using bitewing radiographs	• Diversity • Protection of privacy
3	Lee et al. [6]	Experimental study; (*n* = 304 bitewing radiographs for training CNN model & *n* = 50 for performance evaluation)	Bitewing radiographs (BW)	Deep CNN (U-net) method was used in caries detection. 304 BW radiographs divided into 3 groups; 149 radiographs—structure & CS, 105 radiographs—CS only, 50 radiographs – with no caries	• Diversity (single-centered study)
4	Asker et al. [17]	Experimental study; (*n* = 434 photoraphs (2781 tooth segments) from 51 patients)	Intraoral photographs	CNN (squeezeNet) was used to detect any white spot lesion; fluorotic and non-fluorotic lesions	• Diversity (Single-centered study)
5	Moran et al. [10]	Experimental study; (*n* = 480 different regions of interest from 112 bitewing radiographs)	Bitewing radiographs (BW)	CNN model was used to identify approximal caries using bitewing radiographs and classify them according to severity	• Diversity (Single-centered study) • Accountability (single annotator) • Transparency (with no measure of reliability) • Equity (Difficulty bias) • Protection of Privacy
6	Barayan et al. [18]	Experimental Study; (*n* = 834 bitewing radiographs from 100 patients)	Bitewing radiographs (BW)	ML was used to classify bitewing radiographs	• Diversity (Single-centered study)
**7**	Kuhnisch et al. [5]	Experimental study; (*n* = 2417 intraoral photographs Occlusal surfaces:1317 Smooth surfaces: 1100)	Intra oral photographs	CNN method used for caries detection	• Accountability (single annotator) • Diversity (single-centered study) • Protection of privacy

*RCT* randomized control trial, *CNN* convoluted neural network, *ML* machine learning, *RF* random forest, *RT* random tree, *SVM* support vector machines, *NB* naïve bayes, *CS* caries segmentation.

### Risk of bias

**Fig. 2:**
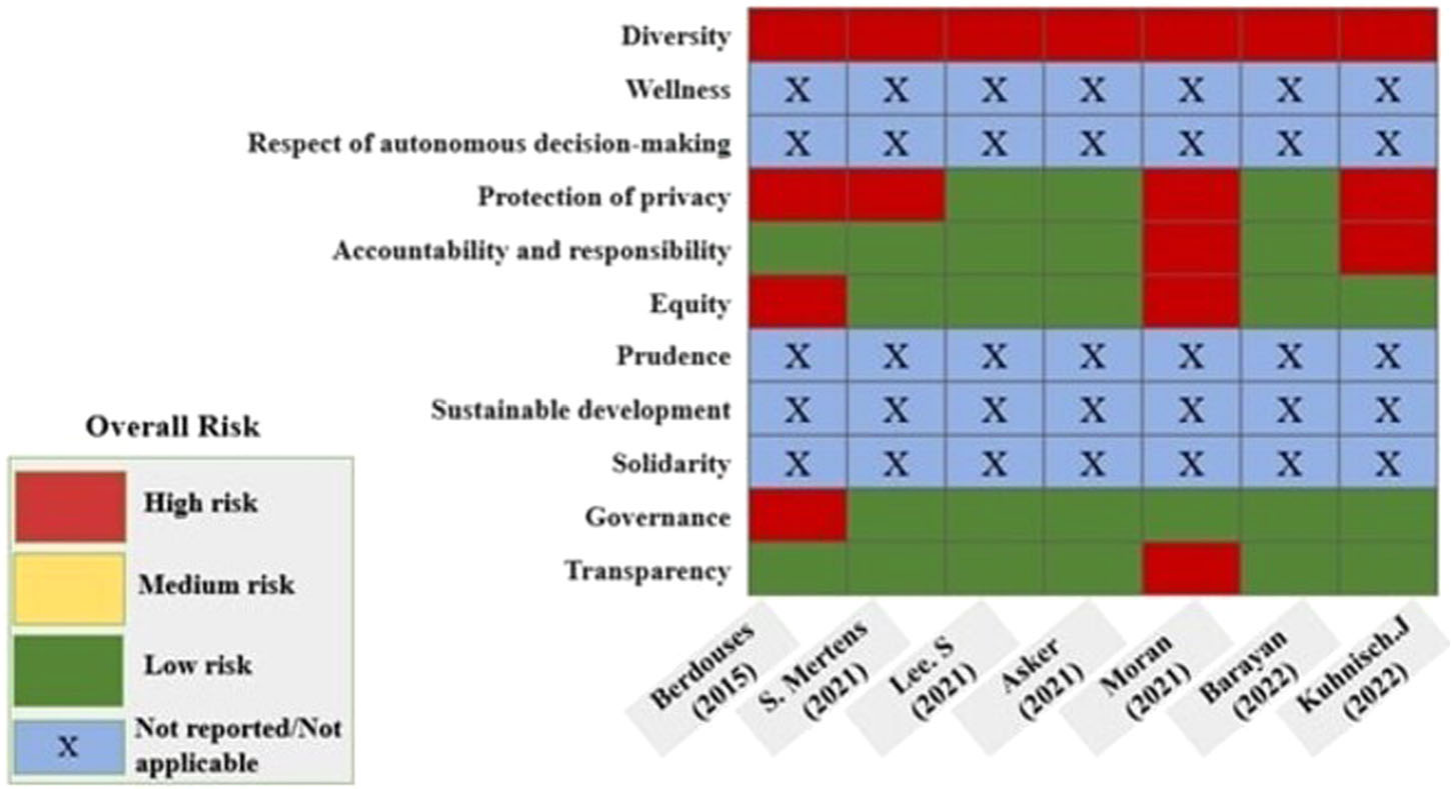
Risk of bias assessment using modified Montreal declaration framework [14].

To assess the risk of bias in each individual study, a modified “Montreal Declaration” tool was designed using the key components of the ethical framework for AI in dentistry [15]. Using this tool each ethical concern was assessed, and studies were categorized as either high risk, moderate risk, low risk and under not applicable category. It was noted that all the studies represented a high risk when the principle of diversity was assessed. Only one study, by Berdouses et al. showed a high risk with respect to governance, since no ethical approval was obtained [19]. The risk of bias assessment of included studies is shown in (Fig. 2).

## Discussion

Unquestionably, the evolution of AI in diagnostic dentistry has been groundbreaking [21]. The current body of evidence has been bustling with newly developed AI models with high performance metrics, anticipating the possibility of a fully automated diagnostic process in the near future [21]. However, ethical regulations surrounding AI remain largely unexplored [21]. As AI-driven decisions increasingly impact dental practitioners and patients, understanding their implications is essential [22]. This paper reframes the discussion on AI in caries detection through an ethical lens, promoting a more inclusive and responsible integration of AI in dental practice.

Across many healthcare sectors, common ethical challenges in AI applications include bias and discrimination, data privacy and security, transparency and accountability, equity and accessibility, and economic and employment effect [23, 24]. For instance, in radiology, AI models rely heavily on imaging data, prompting worries about over-reliance on AI diagnosis [25, 26]. In pathology, assuring the accuracy of AI in diagnosing uncommon diseases and protecting data privacy are critical concerns [27]. In general, the widespread use of AI in patient monitoring and management requires careful consideration of how AI influences patient-doctor interaction [28]. Despite these shared ethical concerns, dentistry presents some unique challenges. Dental care frequently requires personalized treatment plans, making it critical to understand and explain AI suggestions in order to gain patient trust [11, 29]. Additionally, dental radiographs are a fundamental diagnostic tool, needing careful attention to the quality and interpretation of AI-powered radiographic analysis [30]. Furthermore, the possible influence on dental hygienists and technicians must be considered, since AI-driven diagnosis and treatment planning may dramatically modify their responsibilities [31].

AI’s use in dentistry has significant public health benefits, enabling early diagnosis, timely intervention, and improved efficiency [32]. It streamlines diagnostics, reduces errors, and optimizes resource use [32]. Despite these potential benefits, there are significant ethical issues that must be addressed. For instance, the development and use of AI in dentistry must ensure that the technology is equitable and does not reinforce existing prejudices or exclude disadvantaged communities [32, 33]. Furthermore, AI may indirectly impact public health by automating manual jobs, potentially leading to near-term unemployment in low-income communities and adverse health effects due to economic instability [34]. Privacy concerns also arise since AI systems frequently require access to sensitive patient data, demanding strict security measures to ensure patient confidentiality [35]. By recognizing and proactively addressing these ethical challenges, the dental profession may employ AI to improve patient outcomes while maintaining high ethical standards.

To address these ethical challenges, in AI development, regulatory interventions could include transparent processes, promoting inclusivity and diversity, enhancing data privacy, ensuring accountability, and supporting workforce transition [36]. These include clear documentation of data sources, diverse datasets, robust encryption, and clear guidelines for AI-driven decisions [37]. Additionally, training dental professionals to adapt to AI technologies is crucial, ensuring they can effectively use and interpret AI tools [38].

Our review found 7 articles relevant to the objective of this article. Of these, two studies trained the AI models on carrying out automated clinical caries detection, using photographs of carious teeth. The remaining 5 articles focused on radiographic caries detection. Irrespective of the methodology, each article was screened according to the 11 aforementioned ethical principles. The models developed for caries detection are based on deep learning, which has been associated with the “black box effect”—a phenomenon where the internal decision-making processes of the models are not easily understandable to humans [39]. Considering the complex nature of these models, it is often not possible to interpret how a final decision was reached or which component was most important in the decision-making process [40]. This lack of transparency in the decision-making process of the model makes it impossible to determine who can be held accountable for the decisions made by the AI algorithm [41]. At present, the dilemma regarding accountability is further heightened by a gap between those who profit from developments of AI and those who are more likely to deal with the consequences of decisions made by AI [41].

Further widening this gap is the issue of inclusion and diversity [42]. In this context, lack of diversity refers to the inclusion of only one type of ethical or cultural background [22, 42]. The authors found the available literature had a lack of diversity owing to the fact that there were mostly single centered studies from economically developed countries [15]. This geographical bias was also noted by Roche et al. who found that a majority of the literature originated from the US, UK and Germany with limited evidence from low-middle income countries [43]. Ideally, an AI model should be inclusive with respect to gender, social and cultural diversity. Moreover, considering ML algorithms work on pattern recognition by virtue, they may learn biases and discriminate based on age, gender and sexual orientation [44]. This may lead to misdiagnosis due to the AI model’s failure to recognize variations specific to groups based on ethnicity and cultural diversity. Misdiagnosis can lead to false negatives, resulting in untreated caries or false positives, resulting in unneeded treatments and higher healthcare expenditures [45]. However, the current body of evidence is ambiguous regarding the ideal method of incorporating diversity and inclusion while training AI models. This ambiguity is further heightened by the lack of clarity surrounding sample size calculations in AI, making it difficult to determine what percentage of a minority would be sufficient representation to train a model [44]. This is another area that requires further exploration in future studies.

Another area of concern is the issue of patient privacy, since all seven articles did not mention if there was any form of consent obtained by the patient. Moreover, there was limited information on any attempts to conceal identifiers of the patient, which is concerning since several softwares are open to public access. Although it is understandable that it may not be possible to completely de-identify photographs and radiographs, it is pertinent for regulatory bodies to develop a more stringent framework to maintain patient confidentiality and privacy. These “regulatory bodies” encompass research ethics committee which aims to provide thorough, pertinent and prompt evaluations of the research applications across the Faculty of Health Sciences at various institutions. In the selected study, only one study by Berdouses et al. has not reported the process of ethical approval from any ethics committee [19]. However, this may be an ethical concern of publications in general and not specific to AI.

Concerns regarding transparency were also noted in studies by Berdouses et al., since there was only a single annotator present in a study by Kuhnisch et al. and Moran et al., with no measure of the reliability of these annotations [5, 10, 19]. Since labeling the carious defect can often be subjective, it is crucial to calibrate multiple experienced examiners on the process of annotating the defect to avoid any biases. Another noteworthy finding was that Berdouses et al. and Moran et al. both did not mention the random allocation of the included radiographs into the “training set” and “testing set” [10, 19]. This may lead to a difficulty bias, which refers to the possibility of all the challenging sets of images being allocated to either the testing group or the training group.

To date, there is limited evidence on the ethical concerns of AI in dentistry. Recently, an ethical framework and checklist has been developed by Rokhshad et al. [15]. The checklist was devised by a rigorous evaluation process by a carefully selected committee of 29 members [28]. Although this is a commendable initiative, the guidelines were formulated based on existing frameworks for AI, including the World Health Organization’s guidance on ethical considerations in AI for healthcare, and a recently published scoping review on ethics reporting in dentistry [1, 23]. The authors state that although both sources provided valuable insights, neither was entirely applicable to the more specific goals and the context of AI in dentistry.

Although the advancements in AI will greatly advance society, and the benefits of the technology are groundbreaking, it is important to develop a balanced approach to ensure that these benefits are shared equitably and that the growth of AI does not exclude populations that are underrepresented or further heighten any pre-existing bias. With this article, we hope to highlight the requests for transparency in developing AI models. Although this cannot be solved immediately, these factors are important to consider, and there is a need for regulatory bodies to develop adequate frameworks that critically assess the merits and, possibly, the demerits of this dynamic technology.

This article has certain limitations. Due to restricted access, only three databases were searched, which may have limited the identification of relevant studies. Furthermore, most of the included studies were conducted in high-income, single-center settings, potentially impacting the generalizability of the findings. This topographical bias reflects a broader structural disparity—where institutions in well-resourced countries benefit from advanced infrastructure, policy support, and funding, whereas low- and middle-income countries (LMICs) often face significant barriers, including limited digital infrastructure, unequal access to care, and workforce shortages. These differences significantly influence the applicability and successful integration of AI in dental care across regions. Without context-specific adaptations, AI systems developed in high-income settings may fail to address the unique clinical and ethical challenges faced in LMICs.

To bridge this gap, future studies should adopt more collaborative, multicenter approaches across diverse socio-economic settings to ensure broader applicability. Moreover, the notable shortage of literature on AI ethics in dentistry highlights the need for stronger, more targeted primary research. Future investigations must go beyond theoretical discussions and focus on real-world applications—exploring how ethical principles such as transparency, accountability, fairness, and inclusivity are operationalized in clinical dental settings. Empirical studies examining the experiences and perceptions of patients, dental professionals, and AI developers are essential for bridging the gap between ethical theory and clinical application. In addition, to enhance the practical relevance of ethical guidelines, future work should propose concrete, actionable strategies—such as the use of standardized data anonymization techniques (e.g., differential privacy, federated learning) to safeguard patient identity, and the development of diversity inclusion protocols that ensure training datasets represent varied age, ethnicity, and socio-economic backgrounds [46]. Implementation of explainability mechanisms (e.g., saliency maps or decision logs) and mandatory training modules for AI users can also strengthen transparency and accountability [47]. These efforts would support the development of more inclusive, equitable, and context-sensitive AI systems in dentistry, particularly in LMICs.

## Conclusion

Considering the available evidence, the implementation of AI in caries detection is faced with several ethical concerns, including a lack of diversity, protection of privacy and equity, which may perpetuate design flaws within these models. The concerns highlighted in this paper can be addressed by developing a more stringent ethical framework that is specific to AI in dentistry. This is important because in dentistry different AI softwares are required to handle various diagnostic and treatment processes. A robust ethical framework ensures these applications are developed and used responsibly, promoting patient safety, accuracy, fairness, and trust in AI-driven dental care.

## Supplementary information

PRISMA Checklist
